# Bats use social information within and across species

**DOI:** 10.1111/1365-2656.13093

**Published:** 2019-10-09

**Authors:** Antica Culina, Colin J. Garroway

**Affiliations:** ^1^ Netherlands Institute of Ecology NIOO‐KNAW Wageningen Netherlands; ^2^ Department of Biological Sciences University of Manitoba Winnipeg MB Canada

**Keywords:** bats, eavesdropping, echolocation, heterospecific interactions, social information

## Abstract

**In Focus**: LewanzikD., , SundaramurthyA. K., , GoerlitzH. R., (2019). Insectivorous bats integrate social information about species identity, conspecific activity and prey abundance to estimate cost–benefit ratio of interactions. Journal of Animal Ecology, 88, 1462–1473.3094528110.1111/1365-2656.12989PMC6849779

Social interactions can generate social structures that shape the fate of individuals and populations. A key feature of social environments is the information produced by others. Whether actively shared or obtained via ‘eavesdropping’, individuals of many species use publically available information to guide their decision making in important ways. Lewanzik et al. (2019) explore social information use within and across several echolocating bat species. They experimentally manipulated the content of social information about prey abundance with playback experiments of echolocation calls. All species were found to use heterospecific and conspecific social information about conspecific activity levels and prey abundance. This is a rare experimental confirmation of social information use at a community level.

## Graphical abstract

This In Focus article summarizes the approach and the results of an interesting broad‐scale experiment on eavesdropping in bats. The authors discuss the results from a broader perspective of the importance of interspecific community‐level social structure.

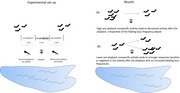

Social interactions, whether competitive or beneficial, shape an individual's environment in ways that affect population and community dynamics, and ultimately evolutionary change (Gil, Hein, Spiegel, Baskett, & Sih, 2018). One particularly important resource for individuals living in social environments is the production of information by other individuals. This information can be transmitted intentionally through activities such as alarm calling, but it is more often unintentionally spread when individuals eavesdrop on the daily activities of neighbours. Animals regularly use such social information to inform important decisions about mate choice, roost or nest site selection, predators and foraging locations (e.g. Aplin, Farine, Morand‐Ferron, & Sheldon, 2012; Dechmann et al., 2009; Doligez, Danchin, & Clobert, 2002). Bats provide a particularly interesting system in which to examine social information use both within and across species for at least two reasons. First, bat species comprise approximately one‐fifth of mammalian species. This means that there will often be a relatively large number of, broadly speaking, trophically similar sympatric species in an area, which provides ample opportunity for eavesdropping. Second, many species from the paraphyletic microbat group echolocate to navigate and to detect their insect prey (Schnitzler, Moss, & Denzinger, 2003). This echolocation is a clear broadcast of specific information pertaining to foraging success. Bats thus provide scope for researchers to directly monitor and manipulate available social information and its use across multiple species. This is exactly what Lewanzik, Sundaramurthy, and Goerlitz (2019) do in their echolocation playback experiment testing of complex patterns of social information use by seven species across 12 lakes in southern Germany.

Insectivorous bats emit high‐pitched echolocation calls while they navigate and forage in 3D space at night, and one would expect that these calls could be used by conspecifics to locate aggregations of insects. Research on such eavesdropping within species has to date yielded somewhat equivocal results (review in Gager, 2019). Even less is known about whether and how information produced by heterospecifics informs foraging decisions, but this too seems likely to happen, especially in species with overlapping diets. Lewanzik et al. (2019) show that bats of three individual species, and one group of four species (that are difficult to distinguish vocally), eavesdrop on conspecific and heterospecific feeding buzzes ‐ the calls emitted while homing in on prey ‐ from six species. Responses to public information were complex and depended on the focal species pair and the background conspecific activity. However, two clear patterns emerged (Figure 1): (a) when conspecific density was high, all species tended to respond to feeding buzzes by reducing their activity; and (b) when conspecific density was low, responses to feeding buzzes were the strongest, regardless of whether species responded by increasing or decreasing activity. In contrast to previous work, Lewaznik et al. controlled for the background activity of bats and presence of conspecifics and heterospecifics. These factors were not controlled for in previous research on eavesdropping in bats—this could explain inconsistencies among previous works.

**Figure 1:**
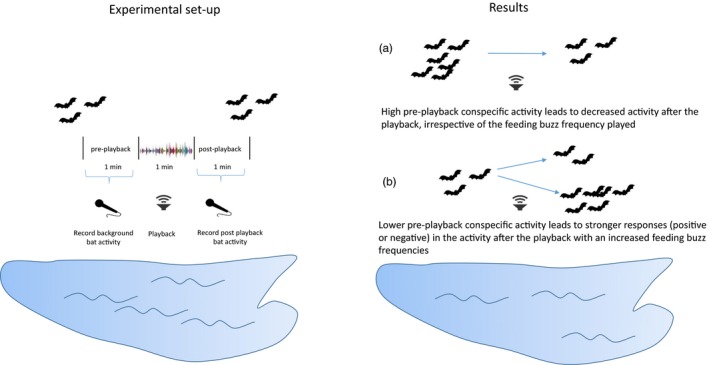
Eavesdropping on social information about prey abundance of three species and one species group of bats on feeding buzz calls from six different bat species was studied using an experimental playback experiment. In the experimental set‐up, applied over 12 central European lakes, 1 min playbacks with different frequency of feeding buzzes (used by bats when they attack their prey) were emitted—more frequent feeding buzzes mimic higher prey density. Bat activity in the area was recorded in the minute before the playback, and in the minute after the playback to determine the change of bat activity as a response to the social signal about the prey density. In all the species pair groups (24 combinations = 6 emitting species x four recipient species), a response to feeding buzz calls was detected, proving that bats eavesdrop on conspecifics and heterospecific calls. These responses were quite complex and depended on a species pair. Yet, two clear patterns emerged, as depicted on the figure: (a) when the conspecific density was high, almost all species responded to feeding buzzes by reducing their activity; (b) when the conspecific density was low, the activity responses (both increase and reduction in activity) to increasing rates of feeding buzzes were generally the strongest

Lewaznik et al. used a well‐designed experiment in natural conditions (Figure 1) to determine whether and how bats use conspecific and heterospecific calls and to explore whether information use depended on the dietary overlap between species and on conspecific density, presumed to reflect level of competition for prey. They used playbacks consisting of search and feeding buzz call sequences of six common species of bats: four Myotis species (Daubenton's, Natterer's, Leisler's bat and long‐fingered bat) and two Pipistrellus species (common and soprano pipistrelles). Playbacks consisted of randomly selected sequences emitted by the species, out of which 0, 12, 24, 48 and 96 were feeding buzzes, each signalling different prey densities. Each playback file started with an empty pre‐playback, followed by a playback with search and buzz calls, followed by an empty post‐playback, each 1 min long. Bat activity in the area was recorded during each of the three phases (pre‐playback, playback and post‐playback). In these recordings, researchers could distinguish between two pipistrelle species (common and soprano pipistrelle) and Daubenton's bats, while four species of genera Nyctalus, Eptesicus and Vespertilio were combined as one group due to similar call structure. The activity of each species or group of species was calculated as number of seconds per minute of playback file in which a species was recorded, including the number of individuals recorded. Based on this, the response variable, the change in activity, was calculated as the difference between responding species activity in the minute after and the minute before the playback. Activity change was then modelled in relation to buzz rate, playback species, conspecifics activity, presence of heterospecifics and time after sunset.

Lewaznik at al. predicted that, when bats eavesdrop to locate food patches, they should be increasingly attracted to the area with simulated higher prey density (i.e. with a higher buzz rate), and this response should be stronger when the dietary overlap with the emitting species is larger. However, the responses to increasing buzz rates should decrease with the increased density of conspecifics, due to competition for limited resources or, perhaps more likely, due to increased auditory clutter. Results of the study confirmed that the majority of bats eavesdrop on feeding buzz calls of conspecifics and heterospecifics, as bats change their activity as a response to feeding buzzes. The direction and the strength of the responses depended on the background activity of conspecifics: at high conspecific densities, almost all species decreased their activity with increasing feeding buzz rates, while at low conspecific densities, the responses to increased feeding buzz rates were usually the strongest (irrespective of whether the response was an increase or decrease in the activity). However, no clear rule emerged as to how dietary or foraging overlap predict species response to buzzes. Based on these results, Lewaznik et al. argue that bats make decisions about the foraging trade‐offs by combining information on foraging overlap and acoustic call structure (as previously suggested, Balcombe & Fenton, 1988; Übernickel, Tschapka, & Kalko, 2013), with the information on prey abundance, conspecifics and heterospecifics activity, preferred foraging habitat and their own competitive abilities.

The authors rightly focus the discussion of their research on the ways in which their work takes our understanding of bat social information use forward. Considering only one species when studying the effects of social information use on population dynamics and space use seems insufficient—responses strongly depend on the overall community of species present. Yet, the potential implications of their findings are broader. Importantly, this work provides rare experimental evidence for the existence of interspecific community‐level social structure. Such social structure should affect the distribution of species in space and time (Goodale, Beauchamp, Magrath, Nieh, & Ruxton, 2010) and, although not examined, evolutionary processes within social groups (Farine, Garroway, & Sheldon, 2012). Increasingly for social species, the social group is being considered an appropriate unit at which conservation and management should be targeted (e.g. Parreira & Chikhi, 2015). If social groups benefit organisms, their breakdown may decrease the likelihood of population resistance. It now seems likely that in some contexts multispecies social structures may be another important level at which conservation and management could be targeted. The foraging success of one species or species group at a site may depend, perhaps reciprocally, on a critical mass of other species. The absence of those species might then cause population declines in other species within the community. To our knowledge, explorations of such community‐level positive density dependence are rare, but this system offers the opportunity for further study of among‐species feedbacks.

## AUTHORS' CONTRIBUTIONS

A.C. and C.J.G. wrote the manuscript and gave the final approval for publication.
